# Gender, sexuality and cis-heteropatriarchal parental control: navigating the politics of neo-panopticon and the resilience of young Khawaja Sara and Hijra in Peshawar, Pakistan

**DOI:** 10.1080/26895269.2023.2299025

**Published:** 2024-01-04

**Authors:** Alamgir Alamgir

**Affiliations:** School of Education, RMIT University Social and Global Studies Centre, Melbourne, Australia

**Keywords:** Cheela, cis-heteropatriarchy, Dera, Guru, Hijra, Khawaja Sara, Pashtun, Panopticon

## Abstract

**Background:**

Scholarly works have extensively explored the marginalized positions of transgender individuals in Pakistan. However, there is a noticeable gap in literature concerning the profound impact of cis-heteropatriarchal parental control on young Khawaja Sara and Hijra individuals—members of the transgender community—particularly when they reside in their parental homes in Peshawar, Pakistan.

**Aim:**

Grounded in Foucault’s panopticon concept, this study seeks to illuminate the pervasive surveillance experienced by young Khawaja Sara and Hijra within the confines of their familial environments.

**Method:**

The study employed a qualitative methodology, which involved conducting face-to-face interviews with 10 members of the Khawaja Sara and Hijra communities in Peshawar.

**Findings:**

The findings demonstrate that trans identities are perceived as a breach of honor in the local Pashtun culture, leading to disrespect and disgrace for the family name. In this way, a meticulous monitoring through regular surveillance creates a neo-panoptic environment within their parental households. This pervasive surveillance not only obstructs their access to quality health services, educational facilities, and employment opportunities but also compels many transgender individuals to abandon their parental homes and migrate to urban areas in Peshawar for their security and protection. Despite grappling with societal pressures, encompassing corporal punishment, sexual abuse, and homelessness, the findings underscore the remarkable resilience and resistance displayed by young Khawaja Sara and Hijra members. Their resilience and resistance serve as a potent challenge against the entrenched cis-heteropatriarchal parental control in Peshawar, Pakistan.

**Conclusion:**

The study concludes on highlighting the harsh circumstances confronted by transgender individuals within their parental households in Peshawar, where their trans subjectivities label them as dishonorable. Despite enduring societal pressures, the resilience exhibited by young Khawaja Sara and Hijra emerges as a formidable challenge to the deeply entrenched cis-heteropatriarchal control. This describes the indomitable strength of the transgender community in overcoming systemic adversities in Peshawar.

## Introduction

The subject of gender and sexuality stands as one of the most highly researched areas in contemporary times, yielding new understandings, ideas, and imaginations regarding human life, identities, and existence. Within these discussions, the inclusion of transgender individuals and their experiences with gender and sexuality in familial dynamics creates a important scholarship to understand their complexities in contemporary societies. This is vital because of the complexities in trans people’s experiences, leading them to struggle in accessing essential quality services for human life and to raise their voices for their rights and advocacy.

This study explores into the intersectionality of gender, sexuality, and the pervasive influence of cis-heteropatriarchal parental control among transgender communities in Peshawar, Pakistan. Its aim is to unveil the layers shaping the lives of transgender children, often relegating them to a marginalized community and out of the mainstream in the society. Previously, researchers in Pakistan extensively examined the marginalized and precarious positionalities of Khawaja Sara and Hijra, which traditionally limited the scope of gender identities and their formation. However, the experiences of transgender individuals in this study navigating societal expectations and in the context of cis-heteropatriarchal parental control in families bring a fresh perspective on the ongoing dialogue on gender and sexuality and trans identities in Pakistan.

The concept of cis-heteropatriarchal parental control explains an ordinary practice that is rooted in traditional gender roles and power dynamics within the Pashtun culture in Peshawar. The influence of this control has significantly impacted the lives of transgender children, shaping their sense of self-discovery and thus are impacting their mental and emotional wellbeing. This study seeks to scrutinize the manifestations of this control and its effects on the lived experiences of transgender children, aiming to illuminate paths toward greater understanding and acceptance using the politics of neo-panopticon in Pashtun Families in Peshawar. I refer to the term “politics of neo-panopticon in Pashtun Families in Peshawar” is a system of power and control within Pashtun families that reflects the concept of a panopticon. In general, the term panopticon explains a typical structure of an institutional building designed to allow a single observer to observe all inmates without them being able to tell whether they are being watched (Foucault, 1977). However, in the context of this study, the concept of “neo-panopticon” suggests a modernized or adapted version of panopticon within Pashtun families in Peshawar. This could imply a system where authority acts and exerts surveillance and control over the individuals, particularly those with transgender subjectivities, influencing their behavior, development, and identities. The term highlights a power dynamic and surveillance mechanism that shapes the experiences of individuals within Pashtun families in Peshawar.

As I embark on this journey into this study, it is imperative to approach trans people and their experiences with sensitivity, empathy, and an open mind. By unraveling these complex threads, I aspire to contribute to a more compassionate and informed discourse surrounding the diverse experiences of transgender children within the broader spectrum of human familial set up.

The terms transgender is used for and by the people whose gender identity or expression does not conform to those typically associated with the sex assigned to them at birth. In South Asia the terms like Hijra^1^ and Khawaja Sara^2^ are used to identify people whose sex assigned to them at birth does not reflect their gender and sexual identity (Dutta & Roy, 2014; Kasmani, 2021; Nisar, 2018; Noor, 2022; Pamment, 2019). Those who are Khawaja Sara and Hijra, when living in their parental houses in Pakistan, are most often subject to frequent surveillance and their daily life activities are closely monitored by elder in their families that limits their choice of living and their gender and sexual identities. Abdullah et al. (2012) found that transgender young people often encounter rejection, stigma, discrimination, and physical abuse within their parental homes, as family members struggle to accept their non-normative behavior. Young Khawaja Sara and Hijra individuals in their early stages of life face abuse in their schools and the wider communities they belong. As they progress into more mature stages of life, they endure both physical beatings and mental torture due to their trans subjectivities, particularly within Pashtun families in Peshawar.

Drawing on the Foucauldian (1975) concept of the panopticon, this article offers a deeper insight into the everyday control and observation of young trans children by their parents who show their transgender characteristics in their parental houses. The analysis is not only focussed on to study the stages and process of socialization but expands the mode of surveillance and regulation that is meant to control and observe the gendered behavior of children in their parental house that lead them in a panoptic situation. In this way, the focus of this article is to demonstrate the parental houses of young Khawaja Sar and Hijra are the neo-panopticon for them when they are living with their families in Peshawar. The findings in this article will show that children with transgender subjectivities are controlled with power and authority by their parents during their cis-heteropatriarchal guardianship, treating and socializing them differently when they express their gender identity and sexuality in Peshawar.

## Methodology and methods: Face to face interviews within the Guru-Cheela houses

A qualitative research methodology was employed, incorporating a combination of face-to-face interviews for data collection with members of the Khawaja Sara and Hijra communities in Peshawar, an urban center in the province of Khyber Pakhtunkhwa, Pakistan. Prior to initiating the data collection process, the project underwent through a comprehensive ethical scrutiny and periodic reviews by the Human Research Ethics Committee at RMIT. This committee is committed and responsible to conduct human research and reflects the values of respect, research merit, integrity, justice, and beneficence.

Therefore, research encompassing both qualitative and quantitative approaches, conducted at RMIT, adheres rigorously predefined ethical standards. Approval from the Human Research Ethics Committee was obtained before commencing the study. A comprehensive set of standards was established to ensure the protection of both the researcher and participants identities and also the well-being of all parties involved throughout the study. These standards included the following features,

### Participants consent

All participants provided informed consent before participating in the study. They were thoroughly briefed on the purpose, procedures, potential risks, and benefits of the research. Participants were assured that their participation was voluntary, and they could withdraw at any point without facing any consequences. All participants were provided a consent form dually signed by both the researcher, participant, and senior supervisor of the study.

### Confidentiality and anonymity

Stringent measures were employed to protect the confidentiality and anonymity of participants. Personal identifiers were removed or replaced with unique pseudonyms to ensure that individual responses could not be traced back to identify or pinpoint any participant. Confidential data were securely stored, and only authorized researchers had access at RMIT system.

### Minimizing the risks

Efforts were made after rigorous consideration to minimize all the potential risks associated with the research. For instance, sensitive topics in the study like sex, sex worker, prostitution were approached with care, and debriefing sessions were offered to participants to address any emotional impact. Participants were asked several times not use term like these that could breach participants’ autonomy and privacy.

### Regular monitoring and review

During the research process a period review was conducted between the research team and by the Human Research Ethics Committee. This ensured that ethical standards were maintained throughout the duration of the study. Any emerging ethical concerns or issues were promptly addressed, and necessary adjustments were made to the research protocol.

## Participants recruitment

For this research study, participants from the Khawaja Sara and Hijra communities were recruited. All the participants were belonged to a diverse group and majority of them had a rural family background, but at the time of interview they all were migrated to Peshawar city. They were residing in city area being a place where they could find security, anonymity, earning opportunities and also finding different ways of living as they wanted to be. The initial step involved an informal meeting with a non-governmental organization that is dedicated to advocating for transgender rights in Peshawar. Through this organization, potential members of the Khawaja Sara communities were notified and invited *via* phone calls or word of mouth for the initial briefing on the project aims and objectives.

Subsequent to the meeting with the NGOs, a separate informal gathering was arranged with the notified individuals from the Khawaja Sara and Hijra communities. During this session, I distributed a flyer outlining the research project. By the end of the meeting, I finalized the list of participants for face-to-face interviews and identified a gatekeeper to assist during the data collection process.

Given the vulnerable nature of the participant groups and the fact that the researcher is a cisgender heterosexual man, the role of the gatekeeper was crucial. The gatekeeper played a key role in facilitating access to the community and establishing a trusting relationship between the researcher and participants. This practice aligns with standard procedures when conducting research with Khawaja Sara communities, as illustrated by Khan et al., who collaborated with a gatekeeper in his study with the Hijras in Bangladesh.

The primary responsibility of the gatekeeper in this study was to disseminate information about the research project within Guru-Cheela networks and provide the researcher’s contact details to potential participants. Subsequently, interested individuals directly contacted the researcher to express their willingness to participate in the research.

## Data collection

The process of data collection was scheduled and completed in the months between May-September 2020. The duration of face-to-face 60 min and all the interviews were properly recorded through audio recorder upon the consent provided by the participants. The interviews were focused on to investigate the parental behavior toward their transgender children in their families and the different ways in regulating trans subjectivities in Peshawar city and also to research the resistance and resilience of young Khawaja Sara and Hijra in their parental houses. At the end of in-depth face to face interviews I came to a position to seek answers for the following striking questions.

1. How Khawaja Sara and Hijra’s parental houses became a neo-panopticon in postcolonial Islamic Pakistan?
2. How parents of young Khawaja Sara or Hijra’s use space in the house to exercise their cis-heteropatriarchal power that are vested in their custodian control over their children in a strict cis-heteronormative familial environment with surveillance and regular monitoring of gender and sexuality.

On completion of the face to face in depth interviews, all the interviews were carefully translated and transcribed and properly coded for further analysis. The stage of translation and transcription was very helpful for developing themes and subthemes and also very crucial for a comprehensive understanding of the content. During translation phase, I converted the original spoken words from Pashto language to English written text. On completing the translation and transcription, I shifted my focus to identify the recurring patterns, ideas, and concepts within the translated and transcribed material. Developing themes involved extracting overarching topics or motifs that encapsulated the main ideas expressed in participants narratives which provided a thematic framework for analysis. In this process, I kept a keen eye for all the details and also understanding of cultural nuances to ensure that the essence of the original content is preserved. By identifying and refining themes, I was able to unlock deeper insights, facilitating a more nuanced and contextually rich interpretation of the original quotes from the data. I recognize that incorporating direct quotes from participants into the text will enhance my narrative, providing an opportunity for their voices to resonate in academic circles worldwide. Utilizing these direct quotes aligns seamlessly with the purpose of this paper.

Moreover, the interpretation of the data assumes a pivotal role in emphasizing the significance of these quotes. I acknowledge that offering insightful analyses of each quote within the text enriches the deeper meaning behind every spoken word. This not only facilitates a more profound understanding of the participants’ narratives but also establishes connections between the quotes and the broader themes and objectives of the paper.

In the case of Pashto quotes, accurate translations are of paramount importance. I have endeavored to provide precise and faithful renditions of every quote in English, ensuring proper attribution to the participants. This approach not only respects the linguistic and cultural diversity of the transgender community in Peshawar but also reinforces the transparency and credibility of my work.

## Results- thematic analysis

This section briefly explains the findings of the study using thematic analysis. The topics examined in detail serve as potent tools for revealing patterns, trends, and underlying meanings inherent in the opinions and feedback shared by the participants. By conducting a systematic and thorough exploration of themes that naturally surfaced from the dataset, I have offered a comprehensive comprehension of the central concepts, recurring motifs, and nuanced perspectives within the study. The following themes describe the pattern of cis-heteropatriarchal parental control in the parental families of Khawaja Sara and Hijra and also demonstrate on the concept of neo panopticon in Peshawar city where young Khawaja Sara and Hijra are oppressed, marginalized and pressurized but at the same time they struggle against the socio-cultural gendered expectation and also raise their voice for their rights and wellbeing.

### Cis-heteropatriarchal parental control- a case of neo panopticon for young Khawaja Sara and Hijra’s

A cis-heteropatriarchal society is specifically one in which the power structures, norms, and expectations are not only organized around traditional gender roles and heterosexuality but also where cisgender individuals are typically favored or privileged over transgender individuals. In such systems, individuals who do not conform to these norms may face challenges and discrimination. For Khawaja Sara and Hijra individuals, navigating a cis-heteropatriarchal parental control environment is always challenging in Peshawar because their identities and understanding always clash with the socio-cultural and religious settings. In this section, I try to demonstrate on the experience and trend of gender identity formation that does not align with the sex assigned to them at birth. This misalignment produce tension in their families and the societies they belong, that are largely rooted in traditional gender roles.

In a cis-heteropatriarchal familial environment, young Khawaja Sara and Hijra face refusal from their families in accepting their gender identity. They are enforced to comply with the societal norms and expectations that further make the living life different for them as they fail to express themselves authentically and the way the understand themselves. This can lead to strained family relationships, misunderstandings, and most often rejection or either they are put under extreme surveillance that develop a panoptic situation in families where parents actively observe and supervise their children behavior and their engagements within their families and outside in the wider communities.

Foucault (1977) in his seminal work describes the idea of panopticon and explores the mode of surveillance and the different means of power regulation within an institution and between the people who have power and those who are powerless. Foucault (1977) views surveillance as a mean to regulate and to ‘fix’ unruly bodies, because for panopticon surveillance is the only reliable tool that makes different ways by watching people directly and to regulate them with power. Therefore, driving Foucault’s understanding of surveillance, power and regulation, this section describes how parents and other members in the families attempt to fix and control the identities of Khawaja Sara and Hijra’s. The tactical process of surveillance and regulation when fails to control the young Khawaja Sara and Hijras, they are forced to leave their parental houses, and relocate themselves to community living known as guru-cheela^3^ houses in Peshawar.

Nargas a guru in her forties discussed in the following way,

As a Khawaja Sara, I’ve always embraced feminine attire, adorned myself with jewellery, and applied lipstick openly whenever possible. We always found it challenging to express our desires openly within our families. Parents, driven by cultural norms, closely monitor their children’s behaviour, leading us to leave our parental houses.

Nargas in this quote discusses two different circumstances of her life. First, she describes her interest in wearing women dresses and jewelleries and to promote herself like females. As she was assigned male, her family members expect her to develop a male oriented behavior in terms of dressing and other familial relationships. But despite all these expectations, she constantly attempted to behave and dresses like girls or females whenever she gets a chance of doing them independently. Here, Nargas traps in two hybrid situations, first, she is expected to behave in gender normative ways and according to the local cultural settings that demand her to do so, and secondly, her own understanding compels her to behave in gendered way as she understands. At this point family expectations and gender understanding comes in continuous clashes that submerge participants’ potential ties within family.

The second part of the quote is about the termination of relationships within families where parents reject their Khawaja Sara and Hijra kids on their trans behavior and they then join the guru-cheela houses. Nargas highlights that she has never been accepted and appreciated for her trans identities within the parental house. She further added that her parents started observing him rigorously and they tried to control her non-normative gendered behavior with power, because the local Pukhtoon culture was not favoring trans behavior. At this point, parental houses become the neo panopticons in contemporary Pakistan because Nargas and many others like her understand themselves differently from the gender that are assigned to them at their birth and in this way, they face the power and regular surveillance in their parental houses. I draw Foucault (1977) concept of ‘docile bodies’ which explains that bodies are the malleable objects for disciplinary actions through power, and where bodies become a medium to inscribe dominant ways of doing things. Here bodies become receptors that can be controlled and disciplined through power and can be modeled to either the category of boy/girl or man/woman to ensure the binary gender system.

Nargas narrative gives explanation to docile bodies as she was trained and socialized by her parents on the family norms and to make her body most likely to be masculinized and to discipline it among the categories of boys or men but when she failed to comply with the instruction then she was manipulated with power. Foucault (1977) described it, “a ‘political anatomy’” and a “mechanics of power” that defined how one may have hold over other bodies, not only so that they may do what one wishes, but so that they may operate as one wishes, with the techniques, the speed and the efficiency that one determines” (138). Thus, a docile body not only becomes a signifier that can be identified as either man or woman but is also meant to be observed easily in the above categories by others.

The third part of the quote is about parental role in socialization which is a common practice in patriarchal families in Peshawar city, where parents and other elder members of the family are responsible to keep an eye on the children’s behavior and their involvement in different indoor/outdoor activities. At this point, the quote is discussing the local Pashtun culture that largely values to shame, honor, and respect. The culture makes the parents responsible to have a regular check on their kids and regularly keep an eye on their children’s activities to observe if they are found in any misconduct that goes against the prevailing socio-cultural setting and where they are behaving differently in terms of their dressing, playing outdoor games, and developing relationships with other members of families. During this course of interplay, initially parents are very humble to their children and try to prevent them from any non-normative gender behavior. Sometimes, children with trans behavior conceal their trans identities because they are afraid of physical punishment and most importantly, they try to hold the honor of their families by not bringing any shame due to their unusual, unacceptable behavior but at some point, the trans kids go against the socio-cultural settings and then they become the point of consideration.

The above discussion demonstrates that young Khawaja Sara and Hijra face serious consequences if they try to live with their trans subjectivities in their parental houses. In this way they are regularly warned by their parent to behave like a cisgender child but if this does not stand workable for their parents, they try to abandon or disown their trans children from their family to keep their family stakes with honor, dignity, and respect. Parents even are in favor of closing their relationships with their trans children because they consider their trans children as a stigma not only for themselves but also for the whole families in the communities they are living. To them, their transgender children bring shame to the family honor and respect. Therefore, they think that abandoning their trans children will save their family honor, and eventually, they will not be ashamed among their own community people.

Similary, Shanza a young cheela talked about here early life activities and the attitude of her family members,

In my early years, I embraced feminine attire, jewellery, and makeup, despite resistance from my sisters. They eventually revealed my transgender identity to my family, leading to pressure to abandon these expressions. My family, viewing me as a boy, imposed restrictions, including physical punishment, and forbade me from engaging in traditionally feminine activities. This control extended to limiting my interactions outside the house due to fears of contact with the Khawaja Sara communities.

Shanza’s narratives mentioned above clearly explain their hidden realities and bitter truths regarding the tough situation they had to go through in their parental houses. Further, participants’ responses also describe the rigid behavior and hostile attitude of family members that were mostly authoritative and meant for taking control over the participants through power and surveillance. Participant further adds that parents and other family members continuously observe their behavior and try to control them using physical violence, calling bad names, detaining them in house, and restricting their meet ups with their friends from Khawaja Sara and Hijra communities.

In addition, Shanza’s narrative further explains Foucault’s (1977) demonstration of power which is always visible and at the same time unverifiable. As discussed in Foucault’s (1977) concept of panopticon, the prisoner becomes self-regulatory and also their own guards in a prison or panoptic structure. Shanza adds that she was warned by her sisters to stop the gendered behavior. This explains that although Shanza’s parents were not present and were not aware because of her gendered behavior, still Shanza was threatened by her sisters who took the charge of mending her ways according to their rules and regulation of surviving in a contemporary society. At this point, parental house became a prison for Shanza where she is threatened to adopt the socio-cultural norms and gender expression.

Family holds a central and revered place within the societal fabric in Pakistan. Families are typically characterized by strong bonds, collective values, and a deep-rooted sense of interconnectedness. At the heart of these familial structures lies the crucial role of parental socialization, a process through which parents impart cultural, moral, and social values to their children. In a country where tradition and heritage play a pivotal role in shaping societal norms, parental socialization becomes a fundamental force in the transmission of cultural identity and societal expectations. This intricate interplay between family dynamics and parental guidance not only influences individual development but also contributes significantly to the preservation and continuity of Pakistan’s rich cultural tapestry. This is further demonstrated by Nayela, a senior guru in guru-cheela houses,

Our parental houses are like a prison for us because we are socialised, raised and cared against the gender expected norms in Pashtun society. We have very limited choice to go against the socio-cultural norms because trans behaviour is considered violation in the families and therefore, we are then punished brutally by our parents and other elders of our families.

From Naylea illustration regarding trans subjectivities and Pashtun parental houses, I can understand that parental house is like a neo panopticon for participants in Peshawar because parents and other family members observe, regulate, and punish the trans behavior of their children. They (parents) do so because the trans performances like wearing feminine dresses, wearing jewelleries, and doing cosmetic makeups by men are not appreciated in Pashtun culture and are thus considered a violation of honor that could bring disgrace and defame to family name. In this way, this is a moral obligation on the individuals in Pakistani societies to become loyal with the family values and hold them (the values) to provide honor, dignity, and respect to other members (Khan, 1997). Failing to do this is considered a violation and can even cost the life under the “honour killing” a regular cultural practice in Pakistan where violators, who fail to keep the values and honor of family, are sentenced to death. Chutti, a young cheela in Peshawar describes that,

I was locked by my parents in a dark room for several days without food and water because of my trans behaviour. Daily I was brutally punished with sticks, rods, and belts. My parents wanted to take a pledge from me to not perform my trans behaviour and do not meet with the members of Khawaja Sara and Hijra communities. My parents warned all members of my family to do not provide any support in any capacity. My parental house was like a prison for me where I was detained/locked in the dark room for months.

Chutti response indicates that the panoptic structure of parental houses drives to control and regulate the gender identity and sexual orientation of young Khawaja Sara and Hijra in their childhood when Parents maintain gender norms and regulate the behavior of their children within the informal supervised structure of panopticism inside the parental houses. In addition, these regulations are not practiced only for power itself, but also for necessities of the dominant cultural belief and other heteronormative practices i.e. family, religion, and socialization.

#### Communal living life: Trans visibility, resilience, and their resistance

Communal living within the communities of Khawaja Sara and Hijra involves understanding of their issues and the challenges they faced due to their gender identity. Participants during the data collection shared that they used to face physical punishment with regular harsh behavior from their family members. But, if these regulations and surveillance do not work for parents in ways as they understand, then they try to abandon or disown their trans child from their family and to keep their family stakes with honor, dignity, and respect. They used to cut their every relationship, because for parents their transgender children are considered a stigma not only for them but for the whole families in the communities where they are living and therefore, they bring shame to the family honor and respect. However, they young Khawaja Sara and Hijra rather than giving up their trans subjectivities they leave their parental houses and join the families of their choice known by guru-cheela houses in Peshawar. Many of the participants discussed that they show resilience against the social pressure they get in their parental houses and in this way every member of the Khawaja Sara and Hijra communities leave their families and comes to guru-cheela houses for living.

Baired writes that trans people with non-conforming gender do not behave or perform in the model gendered norms and then they are faced with punishment upon the violation of gender norms. For Jauk (2013) and Lynch (2005) these punishments and hate violence are the different regulations that are designed to reaffirm the gender norms more likely as binary. Therefore, Foucault (1977) has declared these punishments as the agencies to stabilize and reestablish the norms whenever power passes through individuals.

Travers et al. research on parental support to non-binary children describes that trans youth who are strongly supported in their families always develop higher self-esteem, satisfied life, less depression, low rate of homelessness and better living standard but this was not common in Pashton culture because majority of the participants discussed that they were put in tough situations when their parents and other family members observed their trans characteristics in them.

In this vein, Chocolate, a young cheela in her twenties, responded that,

I identify as a girl or female and strongly align with feminine behaviours. My gender identity leans towards the female spectrum rather than the male. Unfortunately, my family and the community I reside in have consistently rejected this aspect of my identity, adhering strictly to cultural and societal norms that I could never fully embrace. Consequently, I made the decision to distance myself from my parents and other family members, seeking acceptance and understanding in Khawaja Sara or Hijra houses, where my identity is not only acknowledged but also valued.

Chocolate’s narrative clearly explain her hidden realities and bitter truths regarding the tough situation they had to go through in their parental houses. In addition, participants’ responses also describe the rigid behavior and hostile attitude of family members that were mostly authoritative and meant for taking control over the participants through power and surveillance. Participants further added that parents and other family members continuously observe their behavior and try to control them using physical violence’s, calling bad names, detaining them in house, and restricting their meet ups with their friends from Khawaja Sara and Hijra communities that made them vulnerable, and they were unable to demonstrate their self-perceived gender identity and sexual orientation.

Chocolate narrative explains that power is always visible and at the same time unverifiable. As Chocolate added that she was warned by her sisters to stop the gendered behavior. This explains that not only were Chocolate’s parents dissaproving of her being trans, but her sisters too threatened her many times, expecting her to present her gender in ways according to the rules and regulation for survival in a contemporary society.

Foucault (1977) described this as “the principle of their own subjugation”. Thus, panopticon becomes an easy tool to regulate individuals in any institutional setting. Poster (1990) highlighted panopticon as “an imposition of a structure of domination which is meant for controlling masses of people. Similarly, Fiske (1999) observed panopticon is the only structure that could provide ways to efficiently power which is totalitarian and hard to resist. Both Poster and Fiske interpretation of panopticon is justified in the Nayela and Shanza narratives because they discussed that their parents and other member of the family remain dominant on them in their parental houses, and they use power over them for controlling their children and those are hard to resist in the early days of life.

However, despite of participants vulnerability, some responses of Khawaja Sara and Hijras give the impression of resilience, resistance, and refusal that they do in their daily routine life when they move away from their parental houses. The resistance, resilience makes them capable to live in ways as they understand their gender and sexuality. Research on resilience and resistance of transgender communities has neither been studied and under research so far in Pakistan. In this article, Azeema discusses her resilience and the resistance she produced when she was forced out from her parental houses,

When I was compelled to leave my parental home, anxiety gripped me, knowing I had no one on the uncertainties of the streets. Luckily, I found a friend who introduced me to a guru, a senior member of the guru-cheela community, providing a semblance of stability in my tumultuous life. “Za ho deer pareeshana oma kala che ma koor pre hodoo. Ma v oss ba sa k” (Pashto quote). Despite my initial fears that life outside my home would expose me to serious threats, I discovered strength and support among my transgender friends within the guru-cheela houses, ultimately surviving the ordeal (English translation).

Azeema described her story of pain and agony when she left her parental houses because of being trans. Her response showed that she became very vulnerable because she has no money, no food to eat and no room to sleep, but with the support of her friends she resisted against the draconian behavior of her family and now is now living happily within the guru-cheela house where she shares much of her daily routine life with her likeminded friends.

Similarly, Chutti a young cheela who recently joined the guru-cheela houses responded that,

Guru is like mother to us. I consider my guru like my mother and abide all the orders when she gives to me. My guru considers me her real daughter. My guru always supports me when I am in trouble*. Che kala yao sari koor predee awo yawazi she no biya hagha ta support zarorat v* (Direct Pashto quote). Whenever individuals like us leave their families, then they need a support which we get from our guru-cheela relationship (English translation).

Chutti gives an illustration to the living life of Khawaja Sara and Hijra and their relationships with their gurus. This justifies that if a Khawaja Sara and Hijra are forced out from their families, it does not mean that nobody will support them, but they develop their relationships with their senior members in their guru-cheela houses who provided care, love, and affection like the way their mother used to treat their children in their parental families. The shows that for transgender communities in Pakistan relationships have no end, but they are produced new and loveable relationship with their continuous struggle, resistance and resilience that increase the chances of living a beautiful life. In this article, I have found that members of Khawaja Sara and Hijra are regular monitored and continuous surveillance with power and control by their parents and other family members in their parental houses. But when they joined the guru-cheela houses they resist all the negative behavior that they faced from the people in their wider communities and due to their resilience, they are being able to live their life the way they understood their gender and practice their sexuality in their guru-cheela houses despite of all the rigid socio-cultural hegemonic gender and sexual norms and political regulations toward transgender lives in contemporary Pakistan.

## Discussion

In conclusion, the thematic analysis of this study provides a comprehensive understanding of the challenges faced by young Khawaja Sara and Hijra individuals in their parental houses in Peshawar, Pakistan and their resistance and resilience after refusing their parental houses in Peshawar. Drawing on Foucault’s concept of the panopticon, the study illustrates how parents and family members function as central observers, regulating and surveillant the gender expressions and behaviors of Khawaja Sara and Hijra individuals. Within family structures, the pervasive influence of cis-heteropatriarchal parental control creates a neo panopticon, oppressing and marginalizing young trans individuals for their non-normative gender identities.

The analysis accentuates the clash between societal norms and the authentic gender identities of Khawaja Sara and Hijra individuals, resulting in strained family relationships and, in some instances, abandonment. The panoptic nature of parental houses becomes evident as parents wield power and continuous surveillance to control the gender identity and sexual orientation of their children. Participants’ narratives reveal the struggle between the desire for self-expression and the pressure to conform to traditional gender roles, with the looming threat of disownment if they fail to comply.

A critical perspective emerges, emphasizing that the panopticon operates on the principle of normalizing behaviors through the internalization of disciplinary power. For Khawaja Sara and Hijra individuals, societal norms and cultural expectations dictate conforming to traditional gender roles to avoid stigma and discrimination associated with trans subjectivities. The fear of societal discipline compels these individuals to regulate their own behaviors, adhering to established norms even if it contradicts their authentic identities.

In this context, young children bear a moral obligation to remain loyal to their family’s social and cultural values, providing honor, dignity, and respect to other family members (B. Khan, 1997). Failure to uphold these values is considered a violation, risking severe consequences such as “honor killing,” a prevalent cultural practice in Pakistan where violators are sentenced to death for failing to preserve family values.

Foucault also acknowledged the potential for resistance within the panopticon. The study delves into the communal living experiences of Khawaja Sara and Hijra individuals in guru-cheela houses, where they find solace, acceptance, and support. Despite the harsh realities faced in their parental homes, the narratives highlight resilience, resistance, and a refusal to conform to societal expectations. Participants demonstrate the ability to resist disciplinary power, showcasing the potential for societal attitude change toward gender diversity.

## Limitation of the study

This study was conducted with Khawaja Sara and Hijra communities in Peshawar Pakistan, therefore, it is important to acknowledge and address the potential limitations to ensure the study’s findings are interpreted appropriately. As previously described, the communities of Khawaja Sara and Hijra are hard to access because of their trust and security issues, and being an outsider (heterosexual cisgender researcher) in this research it was challenging to mix with transgender communities in Peshawar to get a notable sample of participants from the entire population of Khawaja Sara and Hijra. Therefore, through snowball sampling technique those participants were included in the study who were recommended through connections and agreed to be the part of this study.

Another limitation lies in the geographic scope of this study as it was focused heavily on transgender people in Pashtun culture. Therefore, the findings might not be universally applicable to Khawaja Sara and Hijra residing in other urban centers of Pakistan, including Islamabad, Lahore, and Karachi. The diversity within these regions may result in varied experiences and perspectives not adequately represented by our sample. Lastly, the scope of this project was limited to only the discussing on gender and sexual identity because of high level of ethical consideration. Therefore, in data collection I tried to ensure informed consent of participants, their protection of identities and overall maintaining confidentiality.

## Conclusion

This study contributes to the literature on transgender communities, homelessness, and relocation, shedding light on the resilience and resistance displayed by Khawaja Sara and Hijra individuals. The findings suggest that, with time and continued activism, there is potential for greater acceptance and authenticity within families, challenging cis-heteropatriarchal paradigms and contributing to a more inclusive world for diverse gender expressions. Additionally, the study proposes a new dimension of research, calling for further investigation into transgender relationships within guru-cheela houses. For example, exploring Khawaja Sara and Hijra’s relationships with their like-minded peers and intimate partners is crucial. This is particularly significant because limited literature exists on the intimate relationships of Khawaja Sara and Hijra with their heterosexual cisgender partners and like-minded fellows within their guru-cheela houses in Peshawar.

## Notes

1 The term Hijra is mostly used in India.

2 The term Khawaja Sara is mostly used in Pakistan in addition to the term Hijra.

3 Guru-cheela houses are the places where young Khawaja Sara and Hijra live their senior members after leaving their parental houses.
